# 新诊断原发中枢神经系统弥漫大B细胞淋巴瘤49例临床特征及预后分析

**DOI:** 10.3760/cma.j.issn.0253-2727.2021.11.006

**Published:** 2021-11

**Authors:** 嘉 宋, 惠 刘, 红利 沈, 兰竹 岳, 学军 杨, 文静 宋, 翠云 孙, 士柱 于, 凯 丁, 一浩 王, 丽娟 李, 虹 于, 媛媛 邵, 超盟 王, 树元 岳, 蓉 付

**Affiliations:** 1 天津医科大学总医院血液内科，天津 300052 Department of Hematology, General Hospital of Tianjin Medical University, Tianjin 300052, China; 2 天津医科大学总医院神经外科，天津 300052 Department of Neurosurgery, General Hospital of Tianjin Medical University, Tianjin 300052, China; 3 天津医科大学总医院病理科，天津 300052 Department of Pathology, General Hospital of Tianjin Medical University, Tianjin 300052, China; 4 天津医科大学总医院神经病学研究所，天津 300052 Institute of Neurology, General Hospital of Tianjin Medical University, Tianjin 300052, China

**Keywords:** 中枢神经系统, 淋巴瘤，大B细胞，弥漫性, 布鲁顿酪氨酸激酶, 预后, Central nervous system, Lymphoma, large B cell, diffuse, Bruton's tyrosine kinase, Prognosis

## Abstract

**目的:**

回顾性分析原发中枢神经系统淋巴瘤中弥漫大B细胞淋巴瘤（PCNSL-DLBCL）患者的临床特征及不同治疗方案对患者生存及预后的影响。

**方法:**

回顾性分析天津医科大学总医院自2014年7月至2020年12月收治的49例PCNSL-DLBCL患者的临床资料，根据治疗方案分为MTX组、R-CDOP组、BTKi-R-MTX组和RLZT组，计算各组中位总生存（OS）与无进展生存（PFS）时间，并进行单因素、多因素预后分析，比较各组患者的生存预后。

**结果:**

MTX组、R-CDOP组、BTKi-R-MTX组和RLZT组的中位OS时间分别为16.5个月、4.5个月、42个月和未达到（*P*<0.001），中位PFS时间分别为7个月、1.5个月、20个月和5个月（*P*＝0.005）。多因素预后分析表明是否双表达、IESLG评分和治疗方案是PCNSL-DLBCL患者的预后影响因素。

**结论:**

PCNSL-DLBCL患者的生存预后受治疗方案影响，CD20单抗在PCNSL-DLBCL 治疗中的作用尚有争议，含BTKi的治疗方案有巨大潜力，RLZT方案对于高龄、不耐受大剂量化疗及放疗患者有良好前景。

## Abstract (en)

**Objective:**

The clinical characteristics of patients with primary central nervous system lymphoma-diffuse large B-cell lymphoma (PCNSL-DLBCL) and the effects of different treatment schemes on their survival and prognosis were analyzed retrospectively.

**Methods:**

A total of 49 patients with PCNSL-DLBCL who presented at the Tianjin Medical University General Hospital from July 2014 to December 2020 were included, and their clinical data were retrospectively analyzed. They were divided into four groups: the MTX group, the R-CDOP group, the BTKi-R-MTX group, and the RLZT group. The median overall survival (OS) and progression-free survival (PFS) were calculated, and the survival prognosis was compared by univariate and multivariate prognostic analysis.

**Results:**

The median OS time of the MTX group, the R-CDOP group, the BTKi-R-MTX group, and the RLZT group was 16.5 months, 4.5 months, 42 months, and not reached, respectively (*P*<0.001). The median PFS time of the MTX group, the R-CDOP group, the BTKi-R-MTX group, and the RLZT group was 7 months, 1.5 months, 20 months, and 5 months, respectively (*P*＝0.005). Multivariate prognostic analysis showed that double expressor lymphoma, IESLG risk grade, and different treatment methods were the prognostic factors of PCNSL-DLBCL.

**Conclusion:**

The survival and prognosis of PCNSL-DLBCL are affected by different treatment schemes. The role of CD20 monoclonal antibody in the treatment of PCNSL-DLBCL is still controversial. The treatment scheme containing BTKi has great potential for PCNSL-DLBCL. RLZT scheme has a good prospect for elderly patients who cannot tolerate high-dose chemotherapy and radiotherapy.

原发中枢神经系统淋巴瘤（PCNSL）在所有原发中枢神经系统肿瘤中约占2％，在结外淋巴瘤中占4％～6％[1]。近年来PCNSL的发病率呈上升趋势，诊断PCNSL的中位年龄为65岁[2]–[3]。弥漫大B细胞淋巴瘤（DLBCL）是PCNSL中最常见的病理类型，占90％～95％[4]–[5]。PCNSL-DLBCL具有高度侵袭性，一般累及脑组织、眼部、软脑膜、脊髓等部位。由于其肿瘤特性及累及部位特殊，临床治疗效果不佳，预后差[6]–[7]。本研究回顾性分析了49例PCNSL-DLBCL患者的临床特征并探讨不同治疗方案对患者生存及预后的影响。

## 病例与方法

1. 病例：回顾性分析天津医科大学总医院2014年7月至2020年12月收治的49例新诊断PCNSL-DLBCL患者的临床资料。49例患者中，男32例，女17例，均通过立体定向穿刺技术获得病变组织病理确诊，所有患者的诊断均符合WHO（2008）PCNSL诊断标准，且PET-CT、骨髓细胞形态学和穿刺活检未见体部累及。

2. 治疗方案：49例患者中，单纯化疗38例，化疗联合放疗（全颅脑照射）11例。化疗方案：①甲氨蝶呤（MTX）方案（21例）：MTX 3.5 g·m^−2^·d^−1^，第1天，静脉滴注。部分年龄>65岁患者根据体能状况减量至3.0 g·m^−2^·d^−1^。②R-CDOP方案（8例）：利妥昔单抗375 mg·m^−2^·d^−1^，静脉滴注，第0天；环磷酰胺（CTX）750 mg·m^−2^·d^−1^，静脉滴注，第1天；脂质体阿霉素30 mg·m^−2^·d^−1^，静脉滴注，第1天；长春新碱1.4 mg·m^−2^·d^−1^，静脉滴注，第1天；泼尼松100 mg/d，口服，第1～5天。③布鲁顿酪氨酸激酶抑制剂（BTKi）+MTX+R方案（13例）：伊布替尼560 mg/d，口服，第8～21天；MTX 3.0～3.5 g·m^−2^·d^−1^，静脉滴注，第1天；利妥昔单抗375 mg·m^−2^·d^−1^，静脉滴注，第3天。④RLZT方案（7例）：利妥昔单抗375 mg·m^−2^·d^−1^，静脉滴注，第1天；来那度胺15 mg/d，口服，第1～21天；泽布替尼320 mg/d，口服，第1～21天；替莫唑胺150 mg·m^−2^·d^−1^，口服，第1～5天。如脑脊液检查发现肿瘤细胞，同时予MTX腰椎穿刺鞘内注射治疗。本研究中患者均未接受自体造血干细胞移植和大剂量阿糖胞苷（Ara-C）治疗（因身体状况及患者意愿）。所有患者或患者家属治疗前均知情同意并签署知情同意书，伦理用药经天津医科大学总医院伦理委员会批准（审批号IRB-YX-080-01）。

3. 随访：通过查阅病历或电话的方式进行随访，随访截止日期为2021年3月31日。总生存（OS）时间定义为从初次诊断至死亡或随访终止时间。无进展生存（PFS）时间定义为从首次开始治疗至疾病进展、死亡或随访终止时间。

4. 统计学处理：采用SPSS及Graphpad prism软件进行统计学分析，预后分析采用Kaplan-Meier法，单因素分析采用Log-rank检验，多因素分析采用Cox风险模型，*P*≤0.05为差异有统计学意义。

## 结果

1. 临床特征：49例PCNSL-DLBCL患者中位发病年龄63（33～81）岁，临床表现为头晕、头痛（颅内压升高）患者26例，精神状态改变患者18例，局灶性神经功能缺损患者19例，四肢运动障碍患者20例，癫痫患者3例。单一症状/体征患者18例，两个以上症状/体征患者31例。合并心脑血管疾病患者15例，高血压患者15例，呼吸道疾病患者1例，胃肠道疾病患者11例，糖尿病患者11例，肾病患者2例，风湿性疾病患者3例，肝炎患者2例，皮肤病患者1例，其他实体瘤患者1例。

2. 病灶数量及累及部位 ：49例患者中脑实质受累者46例（其中单一病灶13例，多发病灶33例），脊髓受累3例，眼部受累2例，脑膜受累4例。脑部病灶累及部位：额叶21例，顶叶14例，颞叶17例，枕叶5例，岛叶1例，基底核14例，侧脑室4例，丘脑8例，胼胝体6例，小脑4例，脑干5例。

3. 其他基线特征：男32例，女17例；病理Hans分型：生发中心B细胞（GCB）型10例，非生发中心B细胞（non-GCB）型39例；基因分型：MCD型19例，非MCD型30例；双表达（DEL）15例；TP53阳性6例（共8例检测）；Bcl-2阳性45例，阴性4例；LDH升高17例，正常32例；Ki-67指数≥80％者31例，<80％者18例；美国东部肿瘤协作组（ECOG）评分0分、1分、2分、3分、4分患者分别为2、24、14、8和1例；国际结外淋巴瘤研究组（IESLG）评分低、中、高危患者分别为10、33和6例。

4. 预后因素分析：单因素预后分析显示：性别、年龄（以65岁为界）、D-二聚体水平、合并症种类及数量、脑实质是否受累、病灶数量、ECOG评分等均不是PCNSL-DLBCL不良预后因素。不同Ki-67水平、是否为GCB型的预后差异无统计学意义。而Bcl-2阳性率≥60％、C-myc阳性率≥40％、DEL、IESLG评分高危及不采用含BTKi方案治疗是PCNSL-DLBCL的预后不良因素（表1）。多因素预后分析表明DEL、IESLG评分高危和不采用含BTKi方案治疗是PCNSL-DLBCL的预后不良因素（表2）。

**表1:** 影响49例原发中枢神经系统弥漫大B细胞淋巴瘤患者总生存（OS）的单因素分析

因素	OS［月，*M*（IQR）］	*HR*（95％*CI*）	*P*值
性别			
男	34（12.12）	1.181（0.530～2.629）	0.684
女	15（7.38）		
年龄			
≤65岁	34（9.52）	1.430（0.595～3.437）	0.425
>65岁	16（6.78）		
D-二聚体（µg/L）			
<1000	42（6.61）	1.923（0.867～4.265）	0.107
≥1000	15（4.28）		
合并症数量			
无	15（5.26）	1.445（0.552～3.786）	0.454
1	60（28.14）	0.627（0.251～1.566）	0.317
≥2	34（10.82）		
LDH（U/L）			
<250	36（11.21）	1.763（0.652～3.451）	0.820
≥250	42（13.17）		
是否有脑实质受累			
是	34（10.46）	1.291（0.300～5.558）	0.731
否	15（8.17）		
包块数目			
1	39（13.69）	0.621（0.258～1.498）	0.289
≥2	34（15.59）		
病理类型			
GCB	39（6.33）	1.667（0.655～4.243）	0.284
non-GCB	15（3.98）		
Ki-67			
<85％	42（10.71）	1.870（0.829～4.220）	0.131
≥85	16（6.07）		
Bcl-2 阳性率			
<60％	61（0）	7.159（0.935～54.823）	0.050
≥60％	22（4.42）		
C-myc 阳性率			
<40％	43（15.49）	2.442（1.059～5.632）	0.036
≥40％	15（8.45）		
是否为DEL			
否	43（14.67）	1.338（1.010～1.773）	0.042
是	19（9.24）		
化疗方案			
大剂量MTX	15（4.77）	16.161（2.146～121.715）	0.007
R-CDOP	4（2.12）	38.173（4.732～307.964）	0.001
含BTKi方案	61（29.28）		
是否放疗			
否	22（9.43）	0.663（0.286～1.536）	0.337
是	42（17.53）		
IESLG评分			
低危	39（14.24）	0.187（0.043～0.809）	0.025
中危	22（4.60）	0.295（0.092～0.948）	0.040
高危	6（5.00）		

注：GCB：生发中心B细胞；non-GCB：非生发中心B细胞；DEL：双表达淋巴瘤；MTX：甲氨蝶呤；R-CDOP：利妥昔单抗+环磷酰胺+脂质体阿霉素+长春新碱+泼尼松；BTKi：布鲁顿酪氨酸激酶抑制剂；IESLG：国际结外淋巴瘤研究组

**表2:** 影响49例原发中枢神经系统弥漫大B细胞淋巴瘤患者总生存的多因素分析

因素	*HR*（95％*CI*）	*P*值
性别（男，女）	1.710（0.449～3.058）	0.747
年龄（>65岁，≤65岁）	2.193（0.519～9.271）	0.286
病理类型（GCB，non-GCB）	2.968（0.715～12.327）	0.134
C-myc（<40％，≥40％）	0.619（0.186～2.081）	0.434
是否为DEL		
否	0.457（0.125～1.665）	0.020
是		
化疗方案		
MTX+R-CDOP	40.188（3.800～425.032）	0.002
R-CDOP	24.988（2.338～2667.055）	0.008
含BTKi方案	参照	
IESLG评分		
低危	0.421（0.078～2.282）	0.315
中危	0.264（0.070～0.994）	0.049
高危	参照	

注：GCB：生发中心B细胞；non-GCB：非生发中心B细胞；DEL：双表达淋巴瘤；MTX：甲氨蝶呤；R-CDOP：利妥昔单抗+环磷酰胺+脂质体阿霉素+长春新碱+泼尼松；BTKi：布鲁顿酪氨酸激酶抑制剂

5. 生存分析：至随访截止日期，中位随访时间28（10～108）个月，PCNSL-DLBCL患者22例（44.9％）生存，27例（55.1％）死亡，其中19例因疾病进展死亡，7例因感染并发症死亡，1例在诱导化疗期间因脑疝死亡。

49例PCNSL-DLBCL患者的中位OS时间为18.5（95％*CI* 1～61）个月，2年OS率为34.7％，单纯化疗患者与放化疗联合治疗患者的中位OS时间分别为20.5个月和22.1个月，差异无统计学意义（*P*＝0.130）。49例患者均未行手术切除治疗，MTX组、R-CDOP组、BTKi-R-MTX组和RLZT组的中位OS时间分别为16.5个月、4.5个月、42个月和未达到（*P*<0.001）（图1A）。MTX组、R-CDOP组、BTKi-R-MTX组和RLZT组的中位PFS时间分别为7个月、1.5个月、20个月和5个月（*P*＝0.005）（图1B）。

**图1:**
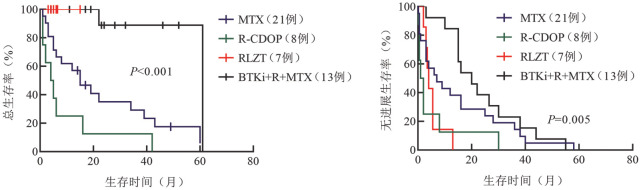
MTX组、R-CDOP组、BTKi-R-MTX组和RLZT组DLBCL患者的总生存（A）及无进展生存（B）曲线 MTX：甲氨蝶呤；R-CDOP：利妥昔单抗+环磷酰胺+脂质体阿霉素+长春新碱+泼尼松；BTKi-R-MTX：伊布替尼+利妥昔单抗+甲氨蝶呤；RLZT:利妥昔单抗+来那度胺+泽布替尼+替莫唑胺

## 讨论

PCNSL是非霍奇金淋巴瘤的一种少见结外类型，老年人多见，其年发病率为0.5/10万[8]。PCNSL 的临床表现不同，主要与受累部位相关。临床上有70％以上患者出现局灶性神经功能缺损，与病灶累及脑实质与软脑膜有关[9]。43％的患者出现非特异性精神改变与行为变化，33％患者出现头痛、恶心、呕吐等颅内压升高表现，14％患者伴癫痫，4％左右患者有眼部受累症状，可表现为视力下降、视物模糊及飞蚊症[10]。本研究组除出现颅内压升高患者比例稍高外，其他基本与文献报道一致。本研究患者均为PCNSL-DLBCL，其中non-GCB型占79.6％，与文献报道相近[11]。

对影响生存的临床特征进行单因素分析发现，Bcl-2表达水平、C-myc表达水平、是否为DEL、IESLG评分等影响患者生存。PCNSL有不同的预后评分系统，主要有基于年龄和karnofsky评分的纪念斯隆凯特琳癌症中心预后模型和IESLG积分系统[12]–[13]。本研究组49例患者按照IESLG 评分分为低危、中危、高危三组，高危是不良预后因素。此外，Bcl-2表达水平、C-myc表达水平、DEL也是本组患者不良预后因素。

Schmitz等[14]提出新的DLBCL基因分型：MCD 亚型（MYD88L265P和CD79B双突变）、BN2 亚型（Bcl-6融合和NOTCH2突变）、N1 型（NOTCH1突变）和EZB 亚型（EZH2突变和Bcl-2易位）。文献报道PCNSL-DLBCL中MCD亚型占37％[15]。MCD亚型较EZB型和BN2型总体预后差[14]。本研究组MCD亚型 19例，占38.8％。MCD亚型依赖B细胞受体信号长期激活，对抑制BCR信号通路的BTKi敏感，适合用BTKi 治疗 [14],[16]。

已有研究报道，放、化疗联合治疗与单纯化疗相比，中位OS的差异无统计学意义[17]。虽然全脑放射治疗对PCNSL的有效率最高可达60％，但缓解持续时间较短，几乎所有患者均复发，中位OS时间仅为 12～18个月，5年OS率仅5％。需要注意的是，全脑放射治疗的远期神经系统不良反应不容忽视，高龄患者发生率可高达58％。本研究组接受颅脑放射治疗的患者神经系统不良反应发生率达75.5％。因此对于PCNSL的一线诱导治疗，放疗仅推荐用于不适合系统性化疗的患者。

PCNSL-DLBCL不治疗者中位生存期仅有1.5～3.3个月[18]，但大剂量MTX（HD-MTX）为基础的化疗方案可以使OS 明显改善，其中位生存期为16.3～66个月[19]–[21]。本研究中MTX组的中位OS时间与其他文献报道的较短中位OS 时间基本一致。本组患者ECOG评分较高，基础合并症多，使OS受到影响。CD20单抗是一种大分子单克隆抗体，理论上不能通过血脑屏障，而且98％小分子药物也不能通过血脑屏障[22]，所以DLBCL经典一线R-CDOP方案对PCNSL-DLBCL疗效欠佳。HOVON 研究显示，在化疗基础上联合利妥昔单抗治疗未改善患者总有效率（ORR）、完全缓解（CR）率、PFS时间和OS时间[23]。IELSG 研究表明，在HD-MTX/Ara-C方案中联合利妥昔单抗可以提高患者ORR，但PFS和OS获益的差异无统计学意义[24]，CD20单抗在PCNSL-DLBCL治疗中的作用尚有争议。

DLBCL发病机制中，BCR信号通路发挥重要信号传导作用，其参与多个下游通路包括NF-κB通路、PI3K/AKT/mTOR通路、MAPK/ERK通路，促进B细胞存活，DLBCL-ABC亚型依赖BCR信号通路[16]。BTKi可以通过血脑屏障，且体外实验证明BTKi与抗DNA合成药物有协同效应[15]。BTKi药物联合化疗治疗初治PCNSL-DLBCL患者的CR率为86％，ORR率为93％，中位OS时间为38个月[15]。复发/难治PCNSL患者接受BTKi联合化疗后CR率为80％，ORR为89％，随访19.7个月，中位OS和PFS时间均未达到[25]。提示BTKi药物对治疗PCNSL具有巨大潜力。

本回顾性研究中还有7例患者采用RLZT方案治疗，尽管观察期尚短，已体现出良好的疗效与安全性、耐受性。有研究表明RLI方案（利妥昔单抗+来那度胺+伊布替尼）对复发/难治PCNSL有良好的临床疗效[26]–[27]。RLZT方案中来那度胺、泽布替尼、替莫唑胺均能透过血脑屏障，且可以产生协同效应，对于高龄、不能耐受大剂量化疗及放疗患者具有良好前景。
